# Exploring the Potential for Collaborative Use of an App-Based Platform for n-of-1 Trials Among Healthcare Professionals That Treat Patients With Insomnia

**DOI:** 10.3389/fpsyt.2020.530995

**Published:** 2020-09-04

**Authors:** Jason R. Bobe, Jessica K. De Freitas, Benjamin S. Glicksberg

**Affiliations:** ^1^Department of Genetic and Genomic Sciences, Icahn School of Medicine at Mount Sinai, New York, NY, United States; ^2^Hasso Plattner Institute for Digital Health at Mount Sinai, Icahn School of Medicine at Mount Sinai, New York, NY, United States

**Keywords:** n-of-1, sleep, clinical informatics, mHealth, RWE, crossover

## Abstract

**Background:**

N-of-1 trials are single patient, multiple crossover, and comparative effectiveness experiments. Despite their rating as “level 1” evidence, they are not routinely used in clinical medicine to evaluate the effectiveness of treatments.

**Objective:**

We explored the potential for implementing a mobile app-based n-of-1 trial platform for collaborative use by clinicians and patients to support data-driven decisions around the treatment of insomnia.

**Methods:**

A survey assessing awareness and utilization of n-of-1 trials was administered to healthcare professionals that frequently treat patients with insomnia at the Icahn School of Medicine at Mount Sinai in New York City.

**Results:**

A total of 45 healthcare professionals completed the survey and were included in the analysis. We found that 64% (29/45) of healthcare professionals surveyed had not heard of n-of-1 trials. After a brief description of these methods, 75% (30/40) of healthcare professionals reported that they are likely or highly likely to use an app-based n-of-1 trial at least once in the next year if the service were free and easy to offer to their patients.

**Conclusions:**

An app-based n-of-1 trials platform might be a valuable tool for clinicians and patients to identify the best treatments for insomnia. The lack of awareness of n-of-1 trials coupled with receptivity to their use suggests that educational interventions may address a current barrier to wider utilization of n-of-1 trials.

## Introduction

Healthcare professionals (HCPs) routinely practice individualized care. They design treatment plans based on unique patient characteristics and clinical presentation, consider various levels of evidence for treatment efficacy, and help patients weigh the risks of side effects and other potential treatment burdens and trade-offs. While there is widespread agreement that we should not expect most treatments to work uniformly across most populations, the systematic evaluation of treatment effect remains a challenge for healthcare professionals and patients (1). Healthcare professionals who practice “evidence-based medicine” generally use comparisons of means and proportions between large groups of patients (e.g., from clinical trials) but are intuitively aware that there exists large heterogeneity of effects for many disease processes and interventions. N-of-1 trials create an opportunity in some contexts for healthcare professionals and patients to individualize treatment selection in a more systematic way. They are designed to help both parties make objective, data-driven treatment choices.

### What Are n-of-1 Trials?

In clinical medicine, n-of-1 trials are used as a decision support tool to inform individualized treatment selection (2). The Oxford Centre for Evidence-Based Medicine ranks n-of-1 trials as “level 1” evidence for determining whether a treatment benefits a patient (3). N-of-1 trials are also viewed as a tool to enhance patient-centered care, insofar as the patient may be involved in the selection of treatments to compare, the selection of outcomes to measure, and the selection of the treatment to continue at the end of the trial (4). Typically, in an n-of-1 trial, a single patient completes a baseline period without any treatments, then alternates between two treatments in a sequence (i.e., “multiple crossover”) (5, 6). Where feasible, treatments may be blinded or placebo-controlled. Outcomes are measured during baseline and each treatment period. At the end of the trial, outcome measurements for each treatment are compared and a treatment is selected. N-of-1 trials may also be deployed to answer other common treatment investigations, such as whether to begin a treatment, proper dosing (7), disease-related nutrition recommendations (8–10), assessing treatment response in people with characteristics (e.g., rare genetic variants) not studied in randomized controlled trials (RCTs) of approved medications (11–13), and deprescribing (14), among others.

To be sure, n-of-1 trials are not useful in every context. They work best in patients with chronic or stable conditions. Non-curative treatments with rapid onset and rapid washout are ideal candidates for n-of-1 trials, whereas treatments with cumulative effectiveness (e.g., some antidepressants) or treatments that disrupt the nature of the underlying condition (e.g., surgery) are not. N-of-1 trials are particularly relevant in contexts where evidence for treatment efficacy is weak or where treatment effects are known to be heterogeneous across populations and among individuals (5).

### Chronic Insomnia: A Testbed for Implementation of n-of-1 Trials

There are many therapeutic contexts where n-of-1 trials are able to serve unmet patient needs. Chronic insomnia is a good target disorder because there is a high prevalence of affected individuals in the general population and also across distinct clinical populations, including Alzheimer’s disease (AD), Parkinson’s disease (PD), attention deficit hyperactivity disorder (ADHD), pregnancy, post-treatment Lyme disease (PTLD) patients (15), and many others.

N-of-1 trials have been implemented or are currently underway in several populations with insomnia. Coxeter et al. reported the effectiveness of valerian versus placebo in 24 patients with chronic insomnia (16). Punja et al. have described a protocol to assess the effectiveness of melatonin in children (ages 6–17) taking medications for ADHD (17). Nikles et al. have described a protocol to assess the effectiveness of melatonin in patients with PD (18).

Many patient populations have insomnia. Patients with insomnia have significant interest in finding the most effective treatment due to the negative impact on quality-of-life for patients, family, and caregivers (19, 20). Poor sleep may also interact with recovery or progression of some diseases, including cardiovascular disease, depression, diabetes, AD, among others (21–25). Estimates of the prevalence of insomnia in the general population vary in part due to the definition of insomnia used. The American Academy of Sleep Medicine (AASM) estimates that 33%–50% of adults experience symptoms of insomnia and 10%–15% of adults experience insomnia disorders that disrupt daily functioning (26).

There is limited efficacy data available for insomnia treatments in many patient populations, so decisional conflict is common. The American Academy of Physicians recommends cognitive behavioral therapy for insomnia (CBT-I) as the first line therapy in adults with chronic insomnia (27). In a systematic review of RCTs that compared CBT-I and medications for the treatment of insomnia, evidence supports the notion that CBT-I is better than medications in some contexts (28). However, among patients that choose to pursue CBT-I, around 20%–30% fail to respond (29). Furthermore, many people do not choose CBT-I because of several treatment-related burdens, including limited access to trained healthcare professionals, weekly therapy appointments, and out-of-pocket costs (30, 31). There is widespread use of pharmacological interventions and over-the-counter (OTC) sleep aids. Around 20% of U.S. adults use prescribed or OTC sleeping medications each month (32). While commonly used, many OTC sleep treatments have limited efficacy and safety data (33). Furthermore, some sleep medications that are commonly used in younger populations for sleep problems are, for example, potentially inappropriate for use in older populations (e.g., hypnotics or Z drugs) (34). For n-of-1 trials to be an effective tool for patients and healthcare professionals, the design of these trials should incorporate the precise needs of the populations they intend to serve.

### Key Questions for the Current Study

Several common themes related to barriers to physician adoption of n-of-1 trials were reported in 2009, based on focus groups with 32 patients and 21 providers in California. First, some clinicians were unclear about the validity of n-of-1 trials. Common concerns raised about cross-over, “sample size” and statistical validity indicates that education in the scientific basis of such trials will be important for adoption. Second, some clinicians were concerned about the potential for n-of-1 trials to interfere with the patient-physician relationship. Third and most germane to the current study, some physicians voiced concerns about the potential time and resource burdens n-of-1 trials would introduce (35). An app-based n-of-1 trial service that is able to reduce some logistical burdens inherent to n-of-1 trials through the automation of previously resource-intensive processes, such as the trial design, study administration and analysis of results, presents an opportunity to potentially enhance adoption of these methods (36, 37).

The purpose of this study is to collect preliminary evidence to inform potential routes for the implementation of an app-based n-of-1 trial platform, called the N1 app. While this platform may be adapted to inform the optimal selection of treatments for a variety of disorders and wellness-related goals, patients with insomnia have been identified as a potential population to target due to the prevalence of the condition, limited evidence for efficacy of treatments across some patient populations, the possibility to incorporate wearable devices for the passive collection of outcome measures (38), among others. With the recent emergence of several app-based tools to support n-of-1 trials generally (39) or self-experimentation with sleep improvement in particular (40, 41), questions about barriers and facilitators to implementation of similar tools for the improvement of patient care or wellness will be of broad interest to the field.

In this study, we aim to assess the familiarity and experience with n-of-1 trials in a convenience sample of healthcare workers that frequently care for patients with insomnia. Our hypothesis is that healthcare professionals are currently familiar with but do not regularly use n-of-1 trials. A secondary aim of the study is to see if there are associations between awareness or utilization of n-of-1 methods and other variables (e.g., age and years in clinical practice). For example, a greater number of years in clinical practice may facilitate exposure to a broader repertoire of clinical methods or lead to the adoption of more traditional clinical practices.

## Methods

### Survey

#### Instrument

The Office for the Protection of Human Subjects at Mount Sinai approved a protocol to administer a voluntary, anonymous survey to healthcare professionals. The exploratory, cross-sectional survey administered has three sections (a) sociodemographics, (b) experience and satisfaction with the treatment of patients with insomnia, and (c) awareness and utilization of n-of-1 trials (see Datasheet S1). The survey was administered online through REDCap (Research Electronic Data Capture) hosted by the Icahn School of Medicine at Mount Sinai (42).

#### Sample and Recruitment

We chose to recruit a convenience sample of clinicians and nurse practitioners through the Department of Psychiatry due to the regularity with which these healthcare professionals treat patients with insomnia. We sent messages through the department mailing list and advertised at bimonthly Grand Rounds events during October and November of 2019.

#### Statistical Analysis

We summarize the survey responses in **Table 1**. We further assessed differences of participants’ awareness of n-of-1 using two-sided Chi-squared test for categorical variables and a t-test for continuous variables which produced p-values and odds ratios. Using the same methods, we also assessed associations for willingness to use a n-of-1 digital service, which we discretized due to low sample size and imbalanced responses into “More likely” (consisting of “Strongly agree” and “Agree” responses) and “Less likely” (consisting of “Neutral”, “Disagree”, and Strongly disagree” responses).

**Table 1:** Sociodemographics of the sample of healthcare professionals surveyed.

Age (mean ± SD)	48.4 ± 16.5
Sex	
Female (n, %)	26 (57.8)
Male (n, %)	18 (40.0)
Not reported (n, %)	1 (2.2)
Race	
Asian (n, %)	3 (6.7)
Black or African-American (n, %)	2 (4.4)
White (n, %)	34 (75.6)
Multiple (n, %)	3 (6.7)
Unknown/Not reported (n, %)	3 (6.7)
Ethnicity	
Hispanic/Latino (n, %)	3 (6.7)
Not Hispanic/Latino (n, %)	41 (91)
Unknown/Not reported (n, %)	1 (2.2)
Clinical degree(s)	
MD (n, %)	39 (86.7)
NP (n, %)	5 (11.1)
MD/NP (n, %)	1 (2.2)
Years of clinical practice	
0–10 (n, %)	20 (44.4)
11–20 (n, %)	9 (20.0)
21–30 (n, %)	7 (15.6)
31–40 (n, %)	5 (11.1)
>40 (n, %)	3 (6.7)
Not reported (n, %)	1 (2.2)
Department(s)	
Psychiatry (n, %)	43 (95.6)
Psychiatry and Anesthesiology (n, %)	1 (2.2)
Not reported (n, %)	1 (2.2)
Primary Specialty	
Psychiatry and Neurology (n, %)	42 (93.3)
Other (n, %)	1 (2.2)
Not reported (n, %)	2 (4.4)

### Ethics Approval

This study has been approved by the Institutional Review Board at the Icahn School of Medicine at Mount Sinai (IRB-18-00789).

## Results

### Survey Results

#### Sample Characteristics

A total of 66 participants consented to the survey, 49 participants completed the survey and the responses from 45 participants were included in the analysis (see **Supplementary Figure 1**). This sample of 45 healthcare professionals from a large, urban hospital are mostly practicing clinicians (88.9%), affiliated with the Department of Psychiatry (88.9%), in their first or second decade of clinical practice (64.4%), with a primary specialty of Psychiatry and Neurology (93.3%) (see **Table 1**).

#### Experience and Satisfaction of Sample With Current Treatments for Patients With Insomnia

Most healthcare professionals surveyed frequently see patients with insomnia in their clinical practice with 88.9% (40/45) reporting daily or weekly encounters. A majority of participants also expressed their dissatisfaction with available treatment options for their patients with insomnia, with 55.6% (25/45) disagreeing or strongly disagreeing with the statement: “I am satisfied with the available treatment options for my patients with insomnia.” Participants also perceived their patients as being dissatisfied with available treatment options, with 62.2% (28/45) disagreeing or strongly disagreeing with the statement: “My patients are satisfied with available treatment options for insomnia” (see **Table 2**).

**Table 2:** Summary of survey results from a sample of healthcare professionals on their experience and satisfaction with current treatments for insomnia.

How often do you see patients in your practice with insomnia?	
Daily (n, %)	17 (37.8)	
Weekly (n, %)	23 (51.1)	
Monthly (n, %)	3 (6.7)	
Quarterly (n, %)	2 (4.4)	
Total (n)	45	
**I am satisfied with the available treatment options for my patients with insomnia**.	
Strongly agree (n, %)	2 (4.4)	
Agree (n, %)	7 (15.6)	
Neutral (n, %)	11 (24.4)	
Disagree (n, %)	21 (46.7)	
Strongly disagree (n, %)	4 (8.9)	
Total (n)	45	
**My patients are satisfied with available treatment options for insomnia**.	
Strongly agree (n, %)	0 (0.0)	
Agree (n, %)	8 (17.8)	
Neutral (n, %)	9 (20.0)	
Disagree (n, %)	24 (53.3)	
Strongly disagree (n, %)	4 (8.9)	
Total (n)	45	
**Have you ever heard of n-of-1 trials?**	
No (n, %)	29 (64.4)	
Yes (n, %)	16 (35.6)	
Total (n)	45	
**How likely are you to use a free service that made it easy for you to offer n-of-1 trials to select patients in your practice with insomnia in order to make data-driven treatment choices at least once in the next year?**	
Highly likely (n, %)	12 (30.0)	
Likely (n, %)	18 (45.0)	
Neutral (n, %)	6 (15.0)	
Unlikely (n, %)	3 (7.5)	
Highly unlikely (n, %)	1 (2.5)	
Total (n)	40	

#### Awareness and Use of n-of-1 trials

Most participants surveyed were unfamiliar with the concept of n-of-1 trials, with 64.4% (29/45) reporting that they had never heard of them before. Following this survey question, participants were presented with a short description of n-of-1 trials that also included a screenshot of the N1 app, a smartphone based n-of-1 trial platform developed at the Icahn School of Medicine (see **Data sheet S1**) (36, 43). They were asked to consider the scenario that “a free service [existed] that made it easy for you to offer n-of-1 trials to select patients in your practice with insomnia. The patient would conduct the mobile app-based trial at home. At the conclusion of the trial, the analyzed results would be available to you and the patient to review together.” Excluding five participants that did not respond to this question, 75% (30/40) of participants reported that they were either “likely” or “highly likely” to “use a service like this to make data-driven treatment choices at least once in the next year.”

Of the 16 healthcare professionals that reported that they were aware of n-of-1 trials, three reported that they had previously used an n-of-1 trial in the treatment of their patients. For the remaining 11 participants that were aware of n-of-1 trials, had never used them in their clinical practice, and reported a primary reason for the lack of adoption, 45.5% (5/11) cited inadequate training in n-of-1 trial design.

We further assessed the relationship between various participant characteristics and having heard of n-of-1 trials (n = 45). We found no significant relationship between having heard of n-of-1 trials and age (p = 0.54, t = −0.61), sex (p = 0.09, χ-squared = 2.91), race (p = 0.10, χ-squared = 7.92), ethnicity (p = 0.75, χ-squared = 0.58), clinical degree (p = 0.84, χ-squared = 0.04; one individual with both degrees not included), number of years practiced (p = 0.29, χ-squared = 5.02), frequency of seeing patients with insomnia (p = 0.50, χ-squared = 2.36), satisfaction of current treatments for insomnia (p = 0.23, χ-squared = 5.65), or perceived patient satisfaction of current treatments for insomnia (p = 0.80, χ-squared = 0.99).

We also assessed the relationship between various participant characteristics and willingness to use a n-of-1 digital service app (n = 40; discretized response). We found no significant relationship between willingness to use a digital app service and age (p = 0.22, t = −1.27), sex (p = 0.23, χ-squared = 1.45), race (p = 0.54, χ-squared = 3.13), ethnicity (p = 0.20, χ-squared = 3.26), clinical degree (p = 0.81, χ-squared = 0.06; one individual with both degrees not included), number of years practiced (p = 0.67, χ-squared = 2.36), frequency of seeing patients with insomnia (p = 0.64, χ-squared = 1.68), satisfaction of current treatments for insomnia (p = 0.21, χ-squared = 5.87), or perceived patient satisfaction of current treatments for insomnia (p = 0.82, χ-squared = 0.93) (see **Table 2**).

## Discussion

The N1 app aims to facilitate the design, administration, and analysis of n-of-1 trials (36, 37). Individuals with insomnia or other chronic sleep disturbance issues are a population that may benefit from access to n-of-1 trials for data-driven treatment selection. While these multi-crossover, comparative effectiveness trials have been in use for decades, awareness, and adoption by healthcare professionals continues to face challenges, as our survey further indicates. Yet, we are encouraged that the healthcare professionals sampled did also report substantial receptivity to future use of app-based n-of-1 trials that were free and enabled collaboration with patients with insomnia who could conduct trials from home.

The lack of awareness of n-of-1 trials coupled with receptivity to their use suggests that educational interventions may address a current barrier to wider utilization of n-of-1 trials. One limitation of this study is the lack of generalizability due to the small sample of healthcare professionals surveyed at a single healthcare system. An important area for future study is to understand how awareness and utilization of n-of-1 trials differs across health systems and other medical specialties that regularly treat patients with insomnia, such as primary care. The survey and analysis conducted here could be deployed in a larger and more diverse sample encompassing providers across multiple specialties and health systems in order to obtain more generalizable knowledge about awareness, utilization, and barriers to adoption of n-of-1 methods and receptivity to the use of an app-based service. The addition of more open-ended questions to the survey, for example, related to barriers to adoption, may also lead to new findings that may not be readily captured in the current instrument. The convenience sampling method used for this survey may also have biased our results due to the possibility that respondents that enrolled were more interested in the idea of app-based n-of-1 trials compared to individuals that did not enroll. A larger and more diverse sample of HCPs may also be able to provide insights on associations between awareness or utilization of n-of-1 trials and other relevant variables, such as years of clinical practice, medical specialty, or age. We found no significant association among the variables we assessed but were also limited by a small sample size. Any such association identified in future studies may help to identify implementation strategies that are tailored for specific subsets of potential end users.

This study also suggests additional work is needed to identify the key barriers and facilitators to implementation in the specific context of an app-based n-of-1 trial service. Prior focus groups identified several key themes among HCPs related to barriers to implementation of n-of-1 methods (35), but that study did not contemplate the potential efficiencies gained or the potential problems introduced through the use of an app-based n-of-1 trial service. The Theoretical Domains Framework (TDF) has been deployed to investigate implementation problems in many healthcare settings, as reviewed in Francis et al. (44). TDF could be used to design interview questions that more thoroughly explores implementation issues related to an app-based n-of-1 trial service with a sample of HCPs and patients in future qualitative research.

While the vast majority of participants expressed a willingness to use an app-based n-of-1 trial platform, we recognize the limitations of this exploratory survey. For example, we do not address the potential complexities involved in getting an app effectively incorporated into existing clinical workflows and training of health care professionals in appropriate use. Moreover, while an app-based platform may reduce some aspects of n-of-1 trials that were previously labor-intensive, such as automated data analysis, new burdens may also be introduced, such as remote end-user support. The engagement of potential end-users, including both patients and providers, to collect more involved feedback about key features and functionality of an app-based n-of-1 trial platform, along with usability testing are important future directions.

Although n-of-1 trials are a powerful tool for patient-centered care in some contexts, we were surprised to find low awareness among our sample of healthcare providers. There may be an important role for organizations such as the Patient-Centered Outcomes Research Institute (PCORI) in the development of guidance materials, or a rubric, that raises the awareness among patients and providers and facilitates implementation of methodologically sound n-of-1 trials.

Historically, several centers have been established to support the implementation of these trials for local clinicians as a service (7, 35, 45). One center currently operates at the University of Queensland in Australia with a focus on sleep (46). While these centers have documented many cases where patients and clinicians were aided in the selection of treatments, the centers are often experiments themselves that last as long as funding permits their operation, for example. For a time in the United States, there was a Current Procedural Terminology (CPT) code for “personalized medicine tests” that suggested a route to reimbursement for the effort required to design, administer, and analyze an n-of-1 trial (4). Writing in 2008, Kravitz et al. suggested that one alternative to the model where n-of-1 trials take root and gain traction primarily through academic clinical centers is the possibility that they “cast off some of their academic trappings and focus on appealing to what patients want and need” (4). Our survey did not address the needs of patients with sleep problems who may benefit from n-of-1 trials. An important future direction is to include a sample of patients with chronic insomnia in qualitative research exploring their perspectives about the use of an app-based n-of-1 trial service for the optimal selection of treatments, either in collaboration with their HCP, or in the case of OTC treatments, through self-guided experiments.

While healthcare practitioners take into consideration each patient’s unique characteristics and strive to use the most up-to-date information to make informed therapeutic recommendations, there will always be some variability and uncertainty in outcomes. Leveraging n-of-1 trials as self-contained experiments can quantify individual outcomes and can better optimize treatment selection in some contexts. We believe growth in the adoption of n-of-1 trials will enhance the precision of treatment selection for many individuals. App-based platforms offer the potential to reduce some barriers, but other challenges still remain.

## Data Availability Statement

The raw survey dataset for this manuscript is not publicly available because of privacy considerations of clinical practitioners.

## Ethics Statement

This study has been approved by the Institutional Review Board at the Icahn School of Medicine at Mount Sinai (IRB-18-00789). The participants provided their written informed consent to participate in this study.

## Author Contributions

All authors listed have made a substantial, direct, and intellectual contribution to the work and approved it for publication.

## Funding

This study is funded by the Icahn School of Medicine at Mount Sinai, a grant from the Steven and Alexandra Cohen Foundation, and a generous gift from Julian Salisbury.

## Conflict of Interest

The authors declare that the research was conducted in the absence of any commercial or financial relationships that could be construed as a potential conflict of interest.
