# Supplementary material for: Exploring the Potential for Collaborative Use of an App-Based Platform for n-of-1 Trials Among Healthcare Professionals That Treat Patients With Insomnia
Source: Front Psychiatry. 2020 Sep 4;11:530995. doi: 10.3389/fpsyt.2020.530995 (PMC7498693; doi:10.3389/fpsyt.2020.530995)
Supplement: Supplementary file 1 [file DataSheet_1.docx]

**Datasheet S1: Survey Instrument**

This 5-minute anonymous survey is for practicing physicians and nurse practitioners affiliated with the Mount Sinai Health System and the Icahn School of Medicine at Mount Sinai.

1. **Are you a practicing physician affiliated with the Mount Sinai Health System or the Icahn School of Medicine at Mount Sinai?**

Yes

No

1. **Are you a practicing nurse practitioner affiliated with the Mount Sinai Health System or the Icahn School of Medicine at Mount Sinai?**

Yes

No

1. **Year of birth (YYYY)**
   __________________________________
2. **Years in clinical practice**

   0-10 years

11-20 years

21-30 years

31-40 years

>40 years

1. **Department(s) you are affiliated with:**

Anesthesiology Perioperative and Pain Medicine Cardiovascular Surgery

Cell Developmental and Regenerative Biology Dentistry

Dermatology

Emergency Medicine

Disaster and Emergency Preparedness Environmental Medicine and Public Health

Family Medicine and Community Health

Genetics and Genomic Sciences

Geriatrics and Palliative Medicine

Health System Design and Global Health Integrative Medicine

Medical Education

Medicine

Neurology

Neuroscience

Neurosurgery

Obstetrics Gynecology and Reproductive Science Ophthalmology

Orthopaedics

Otolaryngology Head and Neck Surgery

Pathology Molecular and Cell Based Medicine Pediatrics

Population Health Science and Policy

Psychiatry

Radiation Oncology

Diagnostic Molecular and Interventional Radiology Rehabilitation and Human Performance

Surgery

Thoracic Surgery

Urology

Other

1. **Primary Specialty**

Allergy and Immunology Anesthesiology

Colon and Rectal Surgery Dermatology

Emergency Medicine

Family Medicine

Internal Medicine

Medical Genetics and Genomics Neurological Surgery

Nuclear Medicine

Obstetrics and Gynecology Ophthalmology

Orthopaedic Surgery Otolaryngology

Pathology

Pediatrics

Physical Medicine and Rehabilitation Plastic Surgery

Preventive Medicine

Psychiatry and Neurology

Radiology

Surgery

Thoracic Surgery

Urology

Other

Not applicable

1. **Race**

American Indian/Alaska Native

Asian

Native Hawaiian or Other Pacific Islander Black or African American

White

More Than One Race

Unknown / Not Reported

1. **Ethnicity**

Hispanic or Latino

NOT Hispanic or Latino

Unknown / Not Reported

1. **Sex**

Female

Male

Other

1. **How often do you see patients in your practice with insomnia?**

Daily

Weekly

Monthly

Quarterly

>3 months between patients

1. **I am satisfied with the available treatment options for my patients with insomnia.**

Strongly agree

Agree

Neutral

Disagree

Strongly disagree

1. **My patients are satisfied with available treatment options for insomnia.**

Strongly agree

Agree

Neutral

Disagree

Strongly disagree

1. **Have you ever heard of n-of-1 trials?**

**They are also sometimes called:**

**- single-patient trials**

**- multi-crossover single-patient trials**

Yes

No

**WHAT ARE N-OF-1 TRIALS?**

N-of-1 trials in clinical medicine are multiple crossover trials, usually randomized, sometimes blinded, conducted in a single patient.

N-of-1 trials are most often used to help clinicians and their patients compare the effectiveness or safety of treatments.

Typically in an n-of-1 trial, a single individual completes a baseline period without any treatments, then alternates between two treatments in a randomized sequence, i.e. 'multiple crossover'.

To-date, more than 2,000 patients have participated in n-of-1 trials that were subsequently published. More than 90% of these patients chose treatments that were consistent with the results of their trials.

With the maturation of digital tools, including validated wearable devices, mobile apps, and electronic patient reported outcomes, there is an opportunity to facilitate much broader patient and clinician engagement in these trials for optimal treatment selection.

[anyone that answered “yes” to item #13, were asked item #14; otherwise this question was skipped]

1. **Have you ever used an n-of-1 trial in the treatment of any of your patients?**

Yes

No

[anyone that answered “yes” to item #14, were asked items #15 and #17; if they answered “no” to item #14, they were asked item #16. Everyone was asked to answer item #18]

1. **How many n-of-1 trials have you ran?**

1

2

3

4

5+

1. **What is the primary reason why you do not use n-of-1 trials in your practice?**

impractical to implement

not sufficiently trained in n-of-1 trial design

too time consuming

not relevant to most patients in my practice

my patients are unlikely to be interested in n-of-1 trials

some other reason

1. **What is the primary reason why you do not use n-of-1 trials in your practice more often?**

impractical to implement

not sufficiently trained in n-of-1 trial design

too time consuming

not relevant to most patients in my practice

my patients are unlikely to be interested in n-of-1 trials

some other reason

1. **Assume that there is a free service that made it easy for you to offer n-of-1 trials to select patients in your practice with insomnia. The patient would conduct the mobile app-based trial at home. At the conclusion of the trial, the analyzed results would be available to you and the patient to review together.**

**How likely are you to use a service like this to make data-driven treatment choices at least once in the next year?**

Highly likely

Likely

Neutral

Unlikely

Very unlikely
