# Supplementary material for: Exploring the Potential for Collaborative Use of an App-Based Platform for n-of-1 Trials Among Healthcare Professionals That Treat Patients With Insomnia
Source: Front Psychiatry. 2020 Sep 4;11:530995. doi: 10.3389/fpsyt.2020.530995 (PMC7498693; doi:10.3389/fpsyt.2020.530995)
Supplement: Supplementary file 2 [file Image_1.pdf]

Supplementary Figure 1.

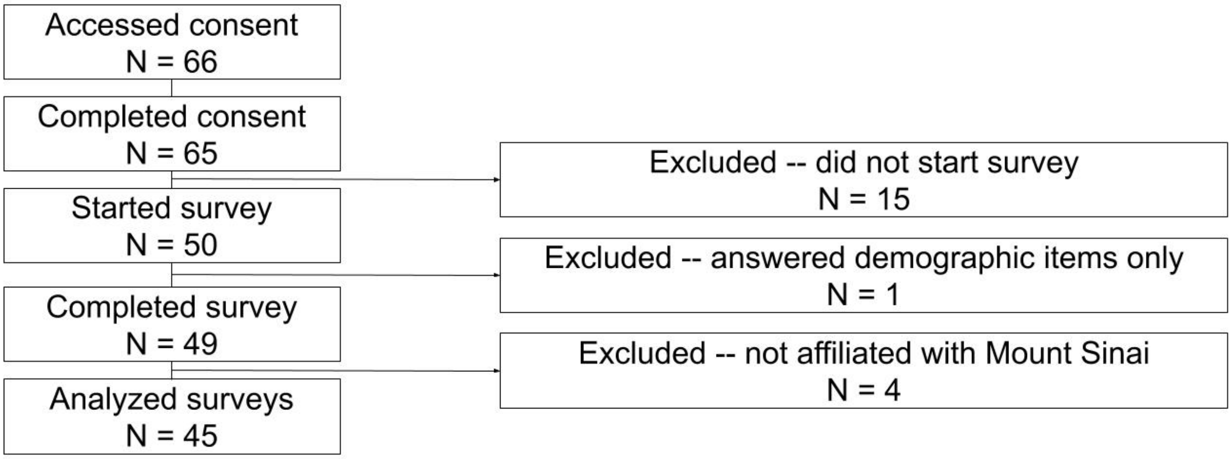

CONSORT diagram for participant flow through the survey, including inclusion and exclusion. Note that we performed quality control and imputed values for two discernable errors (a) One participant (#16) listed their birth year as 1033. We assumed this was a typo. We changed the value to 1933; (b) one participant (#11) skipped question 1 about whether they are an MD. We imputed that their answer is "Yes" based on their answers to subsequent questions, including responses that they are not an NP, they are affiliated with the Department of Psychiatry, their primary specialty as "psychiatry and neurology," and their 21-30 years in clinical practice.
